# Supplementary material for: Quantifying Body Motion Synchrony in Autism Spectrum Disorder Using a Phase Difference Detection Algorithm: Toward a Novel Behavioral Biomarker
Source: Diagnostics (Basel). 2025 May 16;15(10):1268. doi: 10.3390/diagnostics15101268 (PMC12110654; doi:10.3390/diagnostics15101268)
Supplement: Supplementary file 1 [file diagnostics-15-01268-s001.zip › diagnostics-3583321-supplementary.pdf]

Table S1. Results of TD condition

Pair ID	Measurement Period (min:sec.msec)	Density (nods / min)	Mean Phase Difference (ms)	SD (ms)	Kurtosis
1	06:32.8	6.6	180	290	0.9
2	08:16.5	8.4	70	400	0.2
3	09:58.0	8.2	20	300	0.8
4	07:53.6	7.5	30	370	0.5
5	09:16.3	7.7	170	300	0.4
6	06:50.5	12.4	90	340	0
7	07:42.3	8.1	100	240	0.2
8	09:53.8	12.3	90	280	2.6
9	08:38.1	8.1	20	240	1.2
10	09:14.8	7.7	240	330	2.6
11	13:53.8	12.7	90	280	1.4
12	07:47.4	5.5	100	360	-0.2
13	08:18.3	10.7	120	300	0.8
14	08:58.1	17.7	10	240	1.3
15	09:25.5	9.2	60	330	0.7
16	08:55.3	10.2	110	360	0.3
17	08:28.9	14.6	100	250	1.3
18	08:24.9	5.8	20	390	-0.5

Table S2. Results of ASD condition

Pair ID	Measurement Period (min:sec.msec)	Density (nods / min)	Mean Phase Difference (ms)	SD (ms)	Kurtosis
1	08:55.2	2.9	90	410	-0.5
2	14:24.2	6.7	200	400	0.9
3	08:11.6	7.1	90	450	-0.9
4	15:54.8	8.3	0	310	1.5
5	13:17.2	1.9	-30	430	-0.6
6	10:21.0	5.6	100	320	-0.8
7	08:14.6	5.1	110	270	0.9
8	11:41.4	4.1	-10	350	0.2
9	08:24.2	0.3	-580	650	-2.3
10	07:44.2	10.3	150	350	1.4
11	11:16.0	8.4	50	460	-0.4
12	13:42.2	11.3	110	330	1.2
13	08:59.0	6.3	200	350	0.3
14	10:51.8	4.5	230	360	-0.7
15	07:12.3	6.2	290	450	0.1
16	10:26.4	5.1	220	320	0.9
17	09:39.3	7.0	170	320	0.1
18	05:02.7	6.1	130	390	1.0

Table S3. Autism-Spectrum Quotient (AQ) Scores of Speakers and Listeners in the TD and ASD Conditions

Pair ID	TD Condition		ASD Condition	
	TD speaker	TD Listener	TD speaker	ASD Listener
1	10	12	21	30
2	12	15	7	28
3	27	7	22	30
4	13	21	10	21
5	21	17	8	38
6	19	21	28	33
7	17	11	29	35
8	20	24	15	34
9	9	15	13	38
10	19	20	13	36
11	28	16	14	35
12	15	7	26	30
13	6	23	29	10
14	22	7	13	32
15	20	7	19	22
16	16	12	12	37
17	16	20	1	38
18	8	25	7	23

Table S4. Head Movement Density of TD Speakers Across the Two Experimental Conditions (Nods/Min)

Pair ID	TD Condition	ASD Condition
1	20.3	22.8
2	25.5	20.3
3	20.7	14.9
4	21.8	26.5
5	32.5	15.1
6	26.6	29.2
7	26.1	30.6
8	25.2	28.2
9	23.4	31.3
10	21.5	25.1
11	21.4	20.1
12	21.8	30.2
13	26.6	20.1
14	28.5	31.2
15	22.2	18.3
16	17.4	24.9
17	28.2	24.9
18	9.7	23.2

Table S5. Robustness Check of Synchrony Metrics After Excluding Pair 9 from the ASD Condition

Metric	Group Difference (*t*)	*p*-value	Significance
Density	3.49	0.001	Significant
Mean Phase Difference	–1.28	0.210	Not significant
Standard Deviation	–3.14	0.004	Significant
Kurtosis	1.91	0.065	Marginal

Note: These results reflect the group comparisons after excluding Pair 9 from the ASD condition. Findings for density and variability (SD) remained statistically significant, while the group difference in mean phase difference continued to be non-significant, consistent with the original analysis. The marginal result for kurtosis should be interpreted with caution.
